# Effective treatment of post-spinal fusion methicillin-resistant *Staphylococcus aureus* vertebral osteomyelitis with linezolid in a renal-transplant patient

**DOI:** 10.1186/s13104-015-1694-7

**Published:** 2015-11-24

**Authors:** Atsushi Yunde, Kazuhide Inage, Sumihisa Orita, Kazuyo Yamauchi, Miyako Suzuki, Yoshihiro Sakuma, Go Kubota, Yasuhiro Oikawa, Takeshi Sainoh, Jun Sato, Kazuki Fujimoto, Yasuhiro Shiga, Koki Abe, Hirohito Kanamoto, Takane Suzuki, Kazuhisa Takahashi, Seiji Ohtori

**Affiliations:** Department of Orthopaedic Surgery, Graduate School of Medicine, Chiba University, 1-8-1 Inohana Chuo-ku, Chiba City, Chiba 260-8670 Japan; Department of Orthopaedic Surgery, National Hospital Organization, Chiba Medical Center, Chiba, Japan; Department of Orthopaedic Surgery, Chiba Children’s Hospital, Chiba, Japan; Department of Bioenvironmental Medicine, Graduate School of Medicine, Chiba University, Chiba, Japan

**Keywords:** Renal transplant, Linezolid, MRSA vertebral osteomyelitis, Post-spinal fusion

## Abstract

**Background:**

Methicillin-resistant *Staphylococcus aureus* (MRSA)-caused pyogenic spondylitis is a serious complication associated with lumbar fusion surgery. Often, anti-MRSA drugs are not used properly or patients discontinue drug use because of side effects including renal failure.

**Case presentation:**

We report a case at our hospital of a 54-year-old male renal-transplant patient who developed MRSA vertebral osteomyelitis after spinal fusion and was treated effectively with linezolid. After diagnosis of post–fusion surgery osteomyelitis, we conducted emergency flushing and debridement and began linezolid treatment (1200 mg/day, divided) immediately after the surgery. The level of C-reactive protein gradually decreased and became negative 4 weeks after the initiation of linezolid treatment. Serum creatinine level was approximately 1.3 mg/dL throughout the treatment period, indicating no deterioration in renal function.

**Conclusion:**

These results suggest that early flushing and debridement together with linezolid administration is an effective treatment for MRSA vertebral osteomyelitis in renal-transplant patients.

## Background

The use of implants is common in orthopedic surgery but may predispose to postoperative infection, which is a refractory and severe complication. Methicillin-resistant *Staphylococcus aureus* (MRSA) vertebral osteomyelitis is a common infection associated with surgical implants, and is generally treated with anti-MRSA drugs. However, treatment of this disease is difficult because anti-MRSA drugs have poor tissue penetration and are often misused or discontinued because of renal dysfunction, a common side effect. By contrast, linezolid is an anti-MRSA drug with superior tissue penetration that has been shown to be an effective therapy for MRSA osteomyelitis [1]. Furthermore, the pharmacokinetics of linezolid are not influenced by renal dysfunction [2]. Here, we report a case at our hospital of effective linezolid treatment in a renal-transplant patient with post-spinal fusion MRSA vertebral osteomyelitis.

## Case presentation

**Fig. 1:**
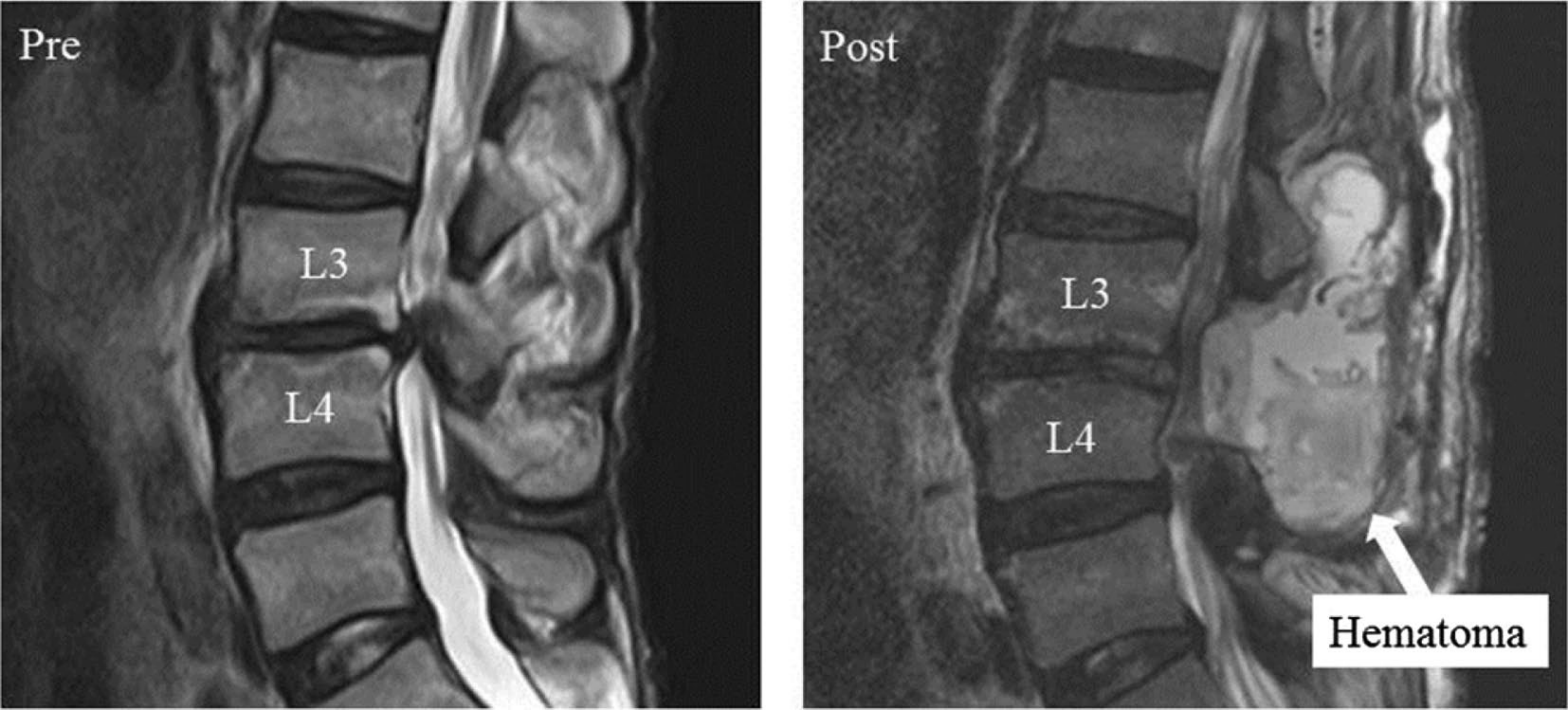
Magnetic resonance imaging of the lumbar spine pre- and post-surgery

A 54-year-old man, who had undergone continuous artificial dialysis for the previous 20 years because of nephronophthisis, underwent a renal transplant at our hospital. After the transplant, he was treated with an immunosuppressive drug (Prograf; 1 mg/day) and a steroid (Predonine; 5 mg/day). The patient was subsequently diagnosed with lumbar spinal-canal stenosis, and strongly requested surgical treatment because of poor quality of life, including lower back pain and intermittent claudication occurring approximately every 5 min. We performed an L3/4 lumbar posterior decompression and posterior lumbar interbody fusion (with use of autogenous bone). The lower back pain was improved after the surgery, but the patient developed a fever 1 week after the surgery. An intensity change in a magnetic resonance imaging (MRI) scan was recognized within the L3/4 intervertebral disk (Fig. 1). An extensive subcutaneous hematoma, which extended into a deep surgical site, was identified. A lumbar puncture of this site was positive for MRSA-positive on bacterial culture, and the patient was diagnosed with post-spinal fusion MRSA vertebral osteomyelitis. At the same time, we checked the linezolid sensitivity, which was positive, and MIC. And it was positive. We therefore performed emergency flushing and debridement and began linezolid treatment (1200 mg/day, divided) immediately after the surgery. However, we did not opt to remove the pedicle screw inserted previously for the purpose of fixation because of the risk of secondary osteoporosis due to long-term systemic use of a steroid.

**Fig. 2:**
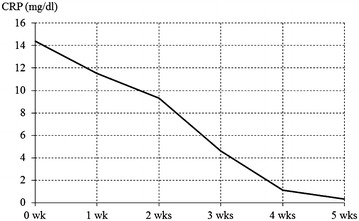
Changes in the serum C-reactive protein (CRP) level

**Fig. 3:**
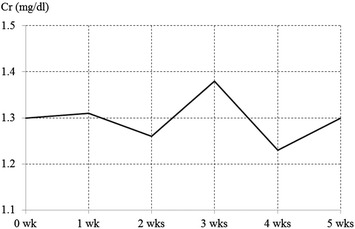
Changes in the serum creatinine (Cr) level

**Fig. 4:**
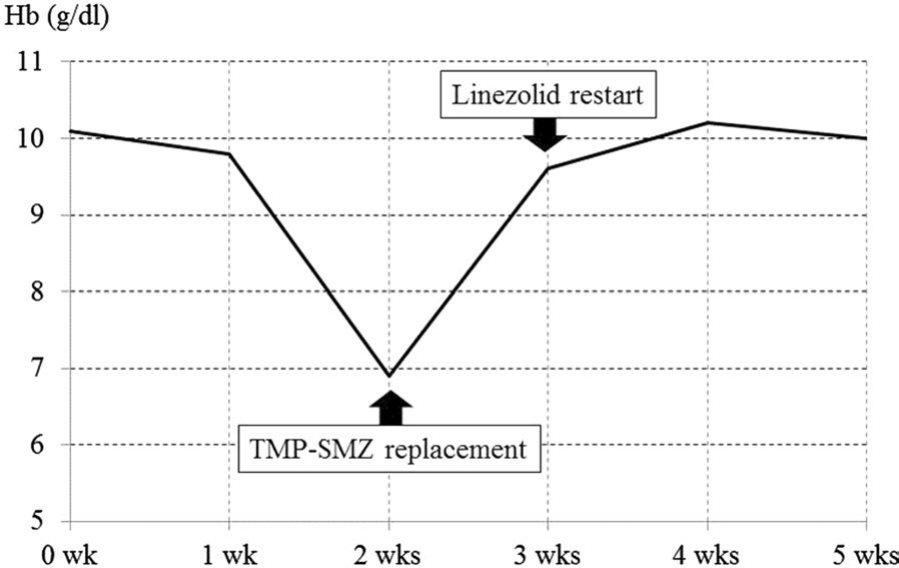
Changes in the serum hemoglobin (Hb) level

Throughout the course of linezolid treatment, the C-reactive protein (CRP) level gradually decreased, becoming negative 4 weeks after administration started (Fig. 2). Serum creatinine (Cr) concentration was approximately 1.3 mg/dL during the treatment period, indicating no deterioration in renal function (Fig. 3). Hemoglobin (Hb) level decreased from approximately 10–6 g/dL within 2 weeks of starting linezolid treatment (Fig. 4), suggesting the development of bone marrow suppression. Fortunately, the infection had been stabilized by early treatment with antibiotics, and linezolid was replaced with a trimethoprim–sulfamethoxazole (TMP–SMZ) combination (320 mg TMP and 1600 mg SMZ) until the Hb level recovered, at which point linezolid use was re-instated. However, we did not administer a preventative antibiotic after the patient tested CRP-negative because of the risk of renal damage. Fortunately, infection has not recurred up to the present time (3 years after the operation).

## Discussion

In many cases of postoperative infection involving surgical implants, it is difficult to remove the implant. According to the guidelines for implant-related MRSA infection published by the Infectious Diseases Society of America (IDSA), use of anti-MRSA drugs is acceptable without removal of the implant only in the case of infection development within 2 months after surgery, implant stability, or sufficient debridement within 3 weeks after the development of infection [3]. In the present case, early flushing and debridement together with linezolid administration was performed to suppress the infection while maintaining the implant. The present case is particularly relevant for secondary osteoporosis patients with long-term systemic use of a steroid. This treatment modality could be used to avoid the risk of aggravation and vertebral instability that comes with removing an implant.

In the present case, we selected linezolid because of its superior tissue penetration and low risk of renal complications. The linezolid in vivo utilization rate in relation to tissue penetration is approximately 100 %, and because linezolid also maintains a good tissue penetration with high water solubility, levels of the drug can reach high concentrations in the bone or cerebrospinal fluid, areas known for poor drug penetration [4, 5]. In the present case, the infection had deeply penetrated the site and the CRP level was 12.5 mg/dL before the start of linezolid administration. CRP level gradually decreased after the initiation of linezolid treatment and eventually became negative 4 weeks following initial treatment. This suggests that linezolid exhibited high tissue penetration. Linezolid is primarily excreted in the urine through its principal metabolites, aminoethoxyacetic acid and hydroxyethyl glycine. The pharmacokinetics of linezolid are not influenced by deterioration of renal function or moderate deterioration of hepatic function, and therefore dose adjustments are not necessary even in the case of renal dysfunction [2]. The present case suggests that linezolid is a valid treatment option because it did not result in deterioration of renal function. However, because some reports describe an increased risk of thrombocytopenia in cases of prolonged linezolid use in patients with deterioration of renal function, patients with renal dysfunction who receive linezolid need to be closely monitored [6].

A common side effect of linezolid use is pancytopenia, caused by bone marrow suppression. Frequency of pancytopenia is significantly increased when linezolid is administered for more than 2 weeks [5]. In cases of pancytopenia, dose reduction or withdrawal of linezolid may be considered, but may be problematic because of the risk of reinfection. In our hospital, dose reduction or withdrawal of linezolid is considered in patients with an Hb level of ≤6.0 g/dL, but if this does not adequately treat the pancytopenia, blood transfusion therapy is an option. Furthermore, according to previous reports, platelet transfusion can be used to treat thrombocytopenia [7]. A decline in Hb level was detected 2 weeks after the start of administration of linezolid in the present case, but because the development of infection had been stabilized, we treated the symptoms by switching treatment from linezolid to a TMP–SMZ combination. These results suggest that early flushing and debridement together with linezolid administration is an effective treatment for post-spinal fusion MRSA vertebral osteomyelitis.

## Conclusion

We describe a renal-transplant patient with post-spinal fusion MRSA vertebral osteomyelitis in whom infection was suppressed without deterioration of renal function by early flushing and debridement together with linezolid administration.

## Consent

Written informed consent was obtained from the patient for publication of this case report and any accompanying images.

## Abbreviations

MRSA: methicillin-resistant *Staphylococcus aureus*
MRI: magnetic resonance imaging
CRP: C-reactive protein
Cr: creatinine
Hb: hemoglobin
TMP–SMZ: trimethoprim–sulfamethoxazole
IDSA: Infectious Diseases Society of America
